# Effects of abdominal hot compresses on indocyanine green elimination – a randomized cross over study in healthy subjects

**DOI:** 10.1186/1471-230X-7-27

**Published:** 2007-07-10

**Authors:** Roman Huber, Sven Weisser, Rainer Luedtke

**Affiliations:** 1 Department of Internal Medicine II, Freiburg University Hospital, Germany; 2 Karl and Veronica Carstens Foundation, Essen, Germany

## Abstract

**Background:**

Hot compresses on the right upper abdomen are used as support for patients with liver diseases in Germany. The study was designed to determine, whether they affect hepatic blood flow.

**Methods:**

Single dose kinetics of indocyanine green (ICG) were studied in 13 healthy subjects with or without hot compresses on the right upper abdomen over 40 minutes. The time interval between the investigations was 8 days, the sequence was randomly assigned.

**Results:**

Within non-linear kinetic analyses the area under the curves (AUC) were 23% (95% confidence interval [CI]: 5–37%) lower with hot compresses. In the initial phase, however, no differences were detected (p = 0.295). The differences occurred only in the late phase after 30–40 minutes, when the genuine ICG is eliminated from the plasma and only the degradation product remains.

**Conclusion:**

Hot compresses have no effect on ICG elimination in healthy subjects but seem to affect the elimination of ICG metabolites.

**Trial registration:**

ClinicalTrials.gov NCT00484913

## Background

Application of hot moist compresses on the upper abdomen is used in nursing care, physiotherapy and rehabilitation clinics as support for patients with liver diseases in Germany [1-4]. Heat is maintained by hot bottles and wrapping the patient in dry cover sheets.

Up to now, there is no scientific evidence whether these applications are effective or whether there is any effect on the liver at all. Aminotransferase levels remained unchanged during abdominal fango treatment of healthy probands and patients with liver disease [5]. In rats the regional hepatic blood flow increased after application of hot peloid paraffin packs on the abdominal wall [6]. It is well known that hepatic blood flow is regulated by the autonomous nerve system. Adrenergic stimulation by norepinephrine decreased the hepatic blood volume in pigs by 35%, vasodilatation increased it by 15% [7]. External heat may reduce the activity of the sympathetic nerve system and therefore increase the hepatic blood volume. Moreover, reflex circuits between the skin and inner organs have been demonstrated in animals and postulated in humans based on anatomical investigations [8,9]. After local and systemic application of heat on the skin without change of the core body temperature, reflectoric vasodilatation in different organs has been described [10,11].

Indocyanine green (ICG) has been used, to demonstrate the effect of various medications on liver blood flow in healthy subjects [12-14]. It is a global liver function test which reflects intrinsic clearance of the liver as well as hepatic blood flow [15]. In healthy subjects, it is mainly determined by the hepatic blood flow [16,17]. The aim of this study was to determine, whether there is an effect of abdominal hot compresses on ICG elimination.

## Methods

Study subjects were recruited among medical students of Freiburg University, Germany. Exclusion criteria were age <18 and >45 years, iodine allergy, any acute or chronic disease [with exception of pollinosis because it is common], elevated levels of alanine amino transferase [ALT], aspartate amino transferase [AST], gamma glutamyltransferase [GGT] or bilirubin, heat urticaria, pregnancy, alcohol intake >30 g/d, any medication except contraceptives and a body mass index (BMI) >30 kg/m^2^.

Subjects were randomized either to be treated with a hot compress or to be left untreated. According to a cross over design the untreated subjects received a hot compress one week later and vice versa.

With each investigation the subjects received a 20 gauge canula in the left and right cubital vein. After 10 minutes rest, 0.3 mg/kg ICG (ICG-PULSION^®^, PULSION Medical Systems AG, Munic, Germany 5 mg/ml) were administered as bolus in the left cubital vein. Blood was drawn from the right cubital vein before ICG application and exactly 3, 6, 9, 12, 15, 18, 21, 30 and 40 minutes after the injection. All samples were centrifuged immediately at 3,500 g for 10 minutes and ICG extinction of serum was measured at 800 nm with a Beckman DU^® ^640 spectrophotometer (Beckman Coulter, USA). From the extinction, ICG half-life and plasma disappearance rate (PDR) were calculated. Furthermore, heart rate, blood pressure, room and skin temperature below the hot compress were measured. All investigations were performed between 8 and 10 am with exactly the same time for each subject. Subjects had to fast over night with the last meal before 10 pm on the day before the investigation. The study was in accordance with the declaration of Helsinki and their extension and was approved by the Ethical Committee of the University Hospital Freiburg. All subjects gave their written consent to participate in the study.

### Application of the hot compress

A twice folded linen sheet (final size 20 × 30 cm) was placed in cooking water and laid on the right upper abdomen as hot as could be tolerated by the subject. Immediately after, a cotton sheet [40 × 45 cm] with 3 pockets for hot bottles was placed on the first sheet. The hot bottles were filled with 400 ml cooking water. A third sheet (50 × 55 cm) was put on the second sheet and thereafter, the subject was wrapped in a 180 × 65 cm cotton sheet. If the bottles were too hot to be tolerated, they could be loosened or removed.

### Statistics

For statistical analyses we fitted two concurring pharmacological models to the data: a one and a two compartment model (backflux or redistribution model) [15]. ICG half life, derived from the one compartment model, was a priori defined as the main outcome parameter; analyses from the two compartment model were regarded secondary. Thus, applying a one compartment model we fitted a log-linear regression line to the ICG extinction data for each subject and each investigation and calculated the ICG half life by dividing the logarithm of 2 with the slope of the estimated regression line. The PDR is the reciprocal of the half time.

A priori power calculations showed that 14 subjects were needed to detect a 0.5 minute difference of half life with a power of 90%. These calculations were based on the assumption that the standard deviation was 0.5 minutes and a one-sample t-test was applied at a 5% level.

In the two compartment model we fitted a biexponential function [a mixture of two simple concurring exponential functions] to the data, again using the logarithmic scale. From this we calculated two concurring half-times [one for each process] and the overall area under the curve [15].

All parameters were analysed by parametric and nonparametric tests following standard techniques for 2 × 2 cross-over-trials [18]. First, we tested for carry-over effects applying a suitable test (two-sample t-test or a Wilcoxon test) for the parameter sums of both investigations. Second, assuming no carry-over effects, we tested for a treatment effect by utilizing the parameter differences from the first to the second investigation.

## Results

14 subjects were recruited for the study. One subject who had erroneously be included with a bilirubin of 1.8 mg/dl had to be excluded from the analysis. Thus, 13 subjects were analyzed. Characteristics of the subjects are shown in table 1. We found no evidence for a carry-over effect from the first to the second investigation (p = 0.474 Wilcoxon test, p = 0.499 t-Test). In the one-compartment model the ICG half-life was 5.88 ± 0.49 minutes without and 5.35 ± 0.53 minutes with hot compresses (Table 2). The difference was significant (p = 0.026 with the non parametric, p = 0.017 with the parametric analysis).

**Table 1:** Characteristics of the subjects [n = 13, means and standard deviations or absolute numbers]

Male/Female	6/7
Age (years)	25.8 ± 4.7
Height (cm)	171 ± 8
Body weight (kg)	65 ± 11
Body mass index (BMI, kg/m^2^)	22.1 ± 2.2
Bilirubin (normal = 1.2 mg/dl)	0.7 ± 0.3
ALT (normal = 50 U/l)	24 ± 15
AST (normal = 50 U/l)	24 ± 8
GGT (normal = 66 U/l)	15 ± 7

ALT: Alanine Aminotransferase; AST: Aspartate Aminotransferase; GGT: Gamma Glutamyltransferase

**Table 2:** Indocyanine (ICG) half-life from the one-compartment model and controlled variables [n = 13, means and standard deviations of 40 minutes investigation]

	without hot compress	with hot compress
ICG-half-life [min]	5.88 ± 0.49	5.35 ± 0.5
k-value (%/min)	11.8 ± 1.0	13.0 ± 1.2
Room temperature [°C]	21.2 ± 0.8	22.1 ± 0.6
Skin temperature right upper abdomen [°C]	32.8 ± 0.9	40.6 ± 1.6
Heart rate [1/min]	68.0 ± 8.4	68.0 ± 11.0
Systolic blood pressure [mmHg]	132 ± 18	132 ± 21
Diastolic blood pressure [mmHg]	86 ± 11	87 ± 16

**Figure 1:**
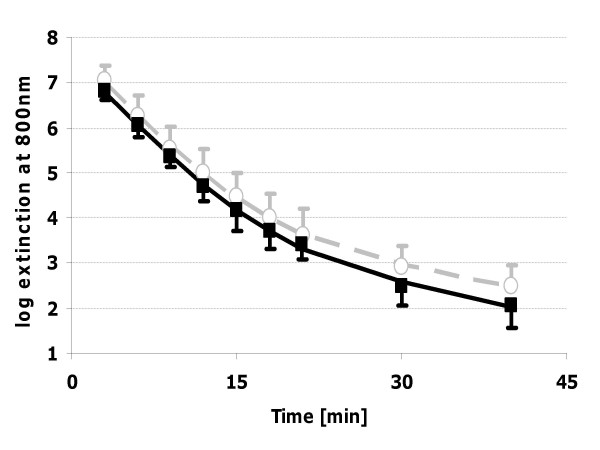
Extinction [logarithmic scale, means ± standard deviation, regression curve, two compartment model] of indoyanine green during the investigations with [■, full line] and without [○, dotted line] hot abdominal compresses.

Within the two compartment model, however, first process half lifes were similar for both conditions (p = 0.295). Second process half lifes were smaller with hot compresses, although not significant (p = 0.127). Figure 1 shows no difference in ICG extinction within the first 21 minutes. In total, the area under the curve (AUC) was 22.8% (95% confidence interval (CI): 5.1–37.3%) smaller with hot compresses (p = 0.0320).

Abdominal skin temperature was 40.6 ± 1.6°C with and 32.8 ± 0.9°C without hot compresses. There were no significant differences in heart rate and blood pressure (Table 2).

## Discussion

Our hypothesis was that abdominal hot compresses might reduce the activity of the sympathetic nerve system and therefore increase hepatic blood flow. Direct effects of heat on the liver are unlikely because of convective heat transport [19,20].

In healthy subjects, ICG elimination has been sensitive to show effects of the circadian rhythm [21], posture [22], food intake [23], age [24] and various medications [12-14,22,25] on the hepatic blood flow. Even if it is normal at baseline, ICG elimination can be further increased [13,25]. The variability of ICG-half life in double experiments has been only 3.9% [26]. Because of high sensitivity and low variability we chose ICG elimination also for our investigations. The setting allowed the subjects to relax. All volunteers reported that the hot compress had been pleasant and most of them fell asleep during the course with hot compress but not during the course without hot compress.

The simplest model [one compartment model] assumes ICG to be distributed only in plasma and hepatic uptake to be a one-way and first order process [15]. Because of the widespread use of this model, we used it for primary analysis and found significant differences in ICG elimination. It has, however, been shown, that a two compartment model, including the temporary redistribution of ICG from the liver to an extrahepatic-extravascular storage much better reflects ICG elimination [15]. We therefore reanalysed the data according to this model. In this analysis no differences in half lifes between both conditions were found, while the total AUCs were significantly different.

This difference in AUCs can be attributed to late ICG elimination because extinction curves showed differences only in late ICG elimination [Figure 1]. Late ICG elimination has not been validated as liver function test. After 30–40 minutes extinction is determined by a metabolite of ICG, which is contained to 1–5% in ICG-PULSION^®^, has a very slow elimination rate, different hepatic membrane carriers and an almost identical absorption spectrum as ICG [15,27]. Therefore, spectrophotometric analysis overestimates late ICG plasma concentrations compared to High-pressure Liquid Chromatography (HPLC) assay methods [28,29]. As degradation of ICG is heat dependent [30], hot compresses might accelerate this process.

ICG elimination does not separate between liver blood flow and liver function so that theoretically an increase in hepatic blood flow may have been missed due to deterioration of liver function. As markers of liver disease (aminotransferase levels, bilirubin) were completely normal and subjects remained healthy during the study, this assumption can, however, be excluded.

As most of the volunteers fell asleep during application of the hot compress, the change in arousal might have interacted with hepatic blood flow. Because synchronized and desynchronized sleep are, at least in animal models, accompanied by no changes or an increase of hepatic blood flow [31,32], it can also be excluded that hepatic blood flow regulation during sleep masked the effect of the hot compress.

In previous, unpublished studies we also failed to demonstrate an effect of hot compresses on portal or hepatic artery blood flow using colour duplex ultrasound. These studies, however, were hampered by strongly artificial conditions (loud noise, breath holding during measurements), which did not allow subjects to relax. Furthermore, measurements strongly fluctuated due to methodological problems (weak signals in hepatic artery, varying positions of the points of measurement during the investigations). By contrast, postprandial changes of portal blood flow can easily be detected with duplex ultrasound.

## Conclusion

Abdominal hot compresses have no effect on hepatic blood flow in healthy subjects with a normal liver. This does, however, not rule out a potential beneficial effect in patients with liver disease.

## Competing interests

The author(s) declare that they have no competing interests.

## Authors' contributions

RH designed the study and supervised the experiments, SW carried out the experiments and RL performed the statistical analysis. All authors read and approved the final manuscript.

## Pre-publication history

The pre-publication history for this paper can be accessed here:


